# Electromyogram in Cigarette Smoking Activity Recognition

**DOI:** 10.3390/signals2010008

**Published:** 2021-02-09

**Authors:** Volkan Senyurek, Masudul Imtiaz, Prajakta Belsare, Stephen Tiffany, Edward Sazonov

**Affiliations:** 1 Geosystems Research Institute, Mississippi State University, Starkville, MS 39759, USA; 2 Department of Electrical and Computer Engineering, Clarkson University, Postdam, NY 13699, USA; 3 Department of Electrical and Computer Engineering, The University of Alabama, Tuscaloosa, AL 35487, USA; 4 Department of Psychology, University at Buffalo, The State University of New York, Buffalo, NY 14260, USA

**Keywords:** Myo, CNN, LSTM, cigarette smoking, wearable sensors

## Abstract

In this study, information from surface electromyogram (sEMG) signals was used to recognize cigarette smoking. The sEMG signals collected from lower arm were used in two different ways: (1) as an individual predictor of smoking activity and (2) as an additional sensor/modality along with the inertial measurement unit (IMU) to augment recognition performance. A convolutional and a recurrent neural network were utilized to recognize smoking-related hand gestures. The model was developed and evaluated with leave-one-subject-out (LOSO) cross-validation on a dataset from 16 subjects who performed ten activities of daily living including smoking. The results show that smoking detection using only sEMG signal achieved an F1-score of 75% in person-independent cross-validation. The combination of sEMG and IMU improved reached the F1-score of 84%, while IMU alone sensor modality was 81%. The study showed that using only sEMG signals would not provide superior cigarette smoking detection performance relative to IMU signals. However, sEMG improved smoking detection results when combined with IMU signals without using an additional device.

## 1. Introduction

According to the World Health Organization (WHO), smoking is a widespread public health problem that has resulted in millions of deaths per year [1]. Smoking causes ten percent of all annual deaths in the world and causes productivity loss, health care expenditures, and other related costs, which correspond to 1.8% of the world’s annual gross domestic product [2,3]. The statistics show the importance of quitting smoking and smoking cessation programs. Understanding the patient’s smoking pattern over time is the primary step of cessation programs. An accurate estimation of smoking activity in an unobstructed way that not only will enhance the efficacy of smoking cessation programs and contribute useful information about smoking behavior, but also enable just-in-time interventions aimed at the control of smoking and reducing smoking-related diseases and deaths [4].

Currently, wearable sensors/devices have attracted attention as a tool for monitoring people’s daily behavioral and physiological activities to observe and understand human behavior and health conditions [5,6]. One of the application areas has been the monitoring of cigarette smoking. Accurate tracking of smoking patterns will not only contribute to our understanding mechanisms underlying smoking, but should also increase the efficacy of our smoking interventions [7,8].

Several studies have evaluated different sensor modalities to automatically detect cigarette smoking in the daily life of smokers [9–13]. One approach has been to measure the respiration pattern. A Respiratory Inductance Plethysmography (RIP) band is a sensor that measures characteristic breathing patterns associated with smoking. The metrics related to smoke inhalation (puff duration, puff volume, and inhale and exhale duration) can be obtained through the analysis of breathing patterns [14]. Measurement of the pattern of breathing, however, is highly sensitive to the motion of hand and body, and challenging for long-term data collection under free-living conditions [15,16].

Another significant approach for the detection of smoking employs motion sensors to detect the signature arm/hand movement associated with a smoking activity. Many researchers have used single or multiple inertial measurement units (IMU) [17–20] worn on the body, such as on the wrist, upper arm, or ankle to detect cigarette smoking activities. A summary of prior studies related to smoking detection via wearable sensors is provided in Table 1.

In [19], arm gestures related to cigarette smoking were detected from two 9-D IMUs (such as the combination of accelerometer, magnetometer, and gyroscope) placed at the wrist and elbow. This study was performed on 15 subjects to collect data on different activities including smoking, eating, drinking, etc. As a predictor of smoking, this study computed the relative position of the elbow to the wrist. A random forest classification model was used to predict motions. For the 10-fold cross-validation, the model accomplished an F1-score of 0.85. The model also accomplished an F1-score of 0.83 when applied to four users wearing IMUs at free living for three days at a rate of 4 h/day.

In [21], four 3-axis accelerometers were placed at the dominant and non-dominant wrist, dominant upper arm, and ankle and detection of both smoking events and puff were reported. This study was performed on six subjects to collect data (11.8 h) on different daily activities including eating, reading, working on a computer and using a phone, etc. For 5-fold cross-validation, the model based on the random forest classifier reported as an F1-score of 0.79 for cigarette smoking detection.

In [22], the authors use a smartwatch on the wrist for detecting smoking activities. They collected 45 h of data from 11 subjects who completed seven daily activities. For a leave-one-subject-out validation, the model reached the F1-score ranging from 0.83 to 0.94.

In [23], four 6-axis IMUs were placed at the dominant wrist and shoulder, and elbows on six participants, and 3.5 h of data was collected from each participant in a controlled laboratory setting. For different participants, the F1-scores between 0.08 and 0.86 were reported.

In [24], a model was proposed employing a RIP sensor to detect inhale–exhale pattern and two IMUs to detect hand gestures. The study was performed on six smokers, which delivered a total of 291 puffs. For 10-fold cross-validation. The support vector-based classifier reached an F1-score of 0.91 for smoking detection.

In our previous study [25], an algorithm was proposed for detection of hand-to-mouth gestures by using single IMU on the wrist and an instrumented lighter. This approach was evaluated from 35 subjects generating a dataset including 55 h from a controlled setting and 816 h from free-living conditions. This support vector machine-based approached achieved an F1-score of 0.86 for puff detection in the controlled environment and 0.85 for the detection of free-living smoking events.

In summary, all previous studies mainly focused on the motion of the forearm to identify hand-to-mouth gestures associated with smoking. However, the motion of the forearm is not sufficient for high accuracy detection of smoking in free-living. Besides, smoking-related gestures are not limited to forearm motions. Many other motions that include finger and hand gestures are also actively engaged during cigarette smoking. Table 2 summarizes possible motions and involved muscles during cigarette smoking [26,27]. Some researchers used multiple IMUs on a different part of the body to obtain higher detection accuracy, such as non-dominant arm, upper-arm, wrist, and chest. Nevertheless, these approaches increase the of devices on users’ body and make the data acquisition more obstructive for long-term usage. This study overcomes this problem by utilizing sEMG signals. By using a single wearable device, both the motion of forearm, via an IMU, and the motion of hand/finger, via sEMG signals, are followed. To the best of our knowledge, usage of sEMG signals have not been investigated for detection of cigarette smoking.

Different sensor approaches were proposed to detect finger and hand movements across a wide range of applications such as sign language translation, human–computer interaction, health care, and virtual reality. Hand data gloves [28,29], inertial rings [30], and Fisheye Rings [31] are wearable sensor systems placed directly at the hand or finger of a user. These wearable sensors are obstructive and not particularly feasible for daily-life activities. Another widely employed approach is the use of surface electromyogram. Rather than tracing the inertial motions of the finger and hand, surface electromyogram is used to sense the electrical representation of the activity of several muscle fibers [27,32,33]. As the activity of most finger and hand muscles can be detected in the forearm, sEMG approaches may be more ergonomically acceptable to users [34].

The current study presents a cigarette smoking recognition method, based on a combination of sEMG and an IMU, deployed in a wearable sensor system. The performance of this method was compared with the method considering individual sEMG and IMU.

## 2. Wearable System and Dataset

### 2.1. Myo^™^ Armband

**Figure 1.**
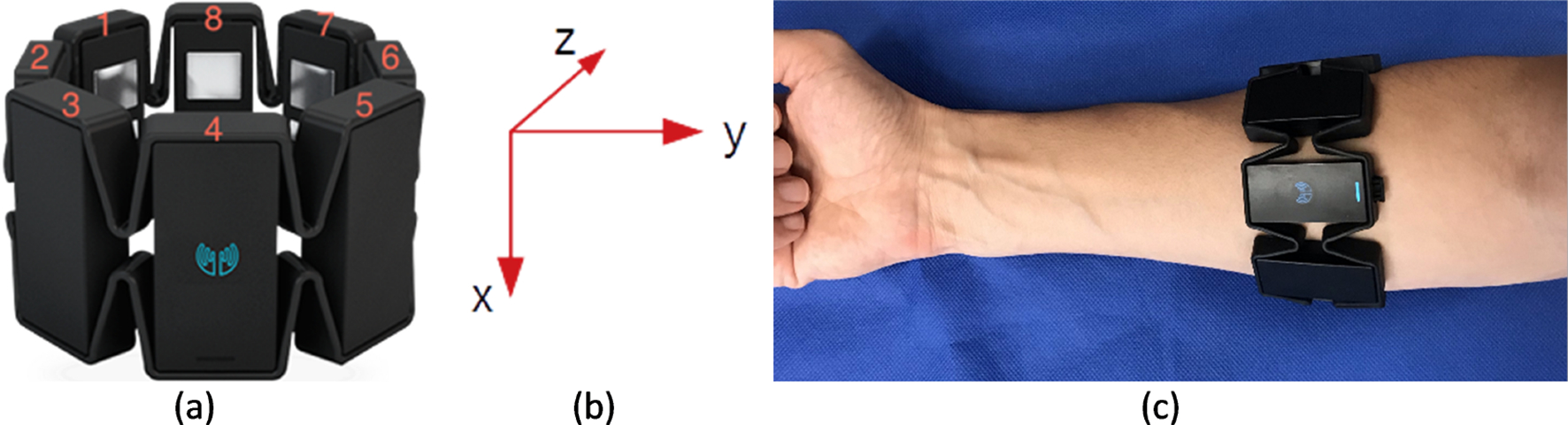
(**a**) Myo armband and eight sEMG channels. (**b**) IMU orientation. (**c**) Myo armband placement on the forearm.

Electromyogram activity and inertial motion of the forearm were recorded using a Myo armband by Thalmic Labs. This low-cost consumer-grade sEMG armband includes eight dry-electrode EMG channels, and a nine-axis IMU (accelerometer, gyroscope, and magnetometer). It can record all sensor channels (with a resolution of 8 bits) simultaneously at a sample rate of 200 Hz. The device uses Bluetooth low energy technology for wireless data transfer. In this study, only EMG, accelerometer, and gyroscope data were used. Figure 1 shows a Myo armband, the orientation of IMU, and device placement on the forearm. With Myo armband placement, as seen in Figure 1c, sEMG signals from superficial muscles of the posterior and anterior forearm can be collected. Superficial muscles of the posterior forearm are extensor carpi radialis brevis, extensor carpi ulnaris, and brachioradialis. The anterior forearm’s superficial muscles are flexor carpi ulnaris, palmaris longus, flexor carpi radialis, and pronator trees. Before employing Myo Armband in the experimentation, the functionality of the Myo device was verified and calibrated by the propriety software and instructions. For this purpose, the propriety software was installed on the computer and synchronized with the Myo armband. A research assistant applied the Myo device to their dominant hands and performed different hand gestures, including grip, hold, swing, etc. The computer software identified the gesture and calibrated accordingly.

### 2.2. Dataset and Study Protocol

**Figure 2.**
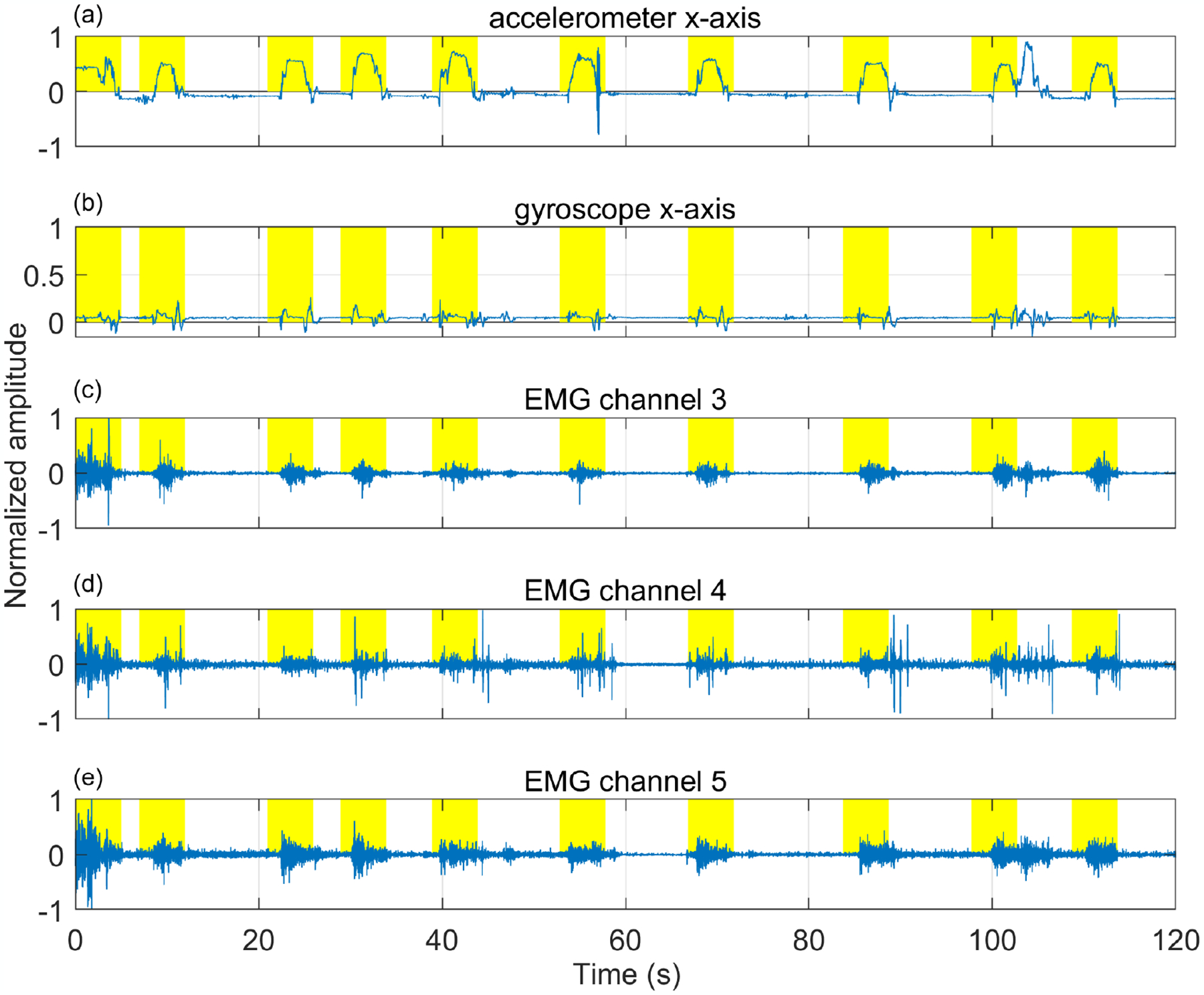
An example of sensor signals from a participant while sitting and smoking. (**a**) accelerometer x-axis, (**b**) gyroscope x-axis, (**c**) EMG channel-3, (**d**) EMG channel-4, and (**e**) EMG channel-5. Highlighted areas show puffing events.

The dataset used in this study is a part of the PACT 2.0 [35] validation study, which includes 35 participants. Those smokers were allowed to participate in this study who had a history of smoking for at least one year and carbon monoxide (CO) levels >8 ppm. However, 19 subjects’ data were excluded because of missing data during wireless data transfer and noise due to Myo armband’s loosening. The remaining 16 subjects (male: 9, female: 7; 9 Caucasian, 3 Asian, 2 American Indian, and 2 African American) had an average age of 22.5 ± 5.6 years, average smoking history of 6.23 ± 7.25 years, average Body mass index of 24.6 ± 6.12 kg/m^2^, an average weight of 73.9 ± 17.64 kg, and an average chest circumference of 85.63 ± 12.34 cm. The subjects’ self-reported cigarette consumption was 11 ± 5.54 per day, and with an average CO level of 14 ± 6.21 ppm measured during the screening. Each subject was pre-aware of the study protocol which approved by the Institutional Review Board of the University of Alabama. Each participants provided their consent for participation in the written format. Subjects reported that they were healthy and had no acute or chronic respiratory problems. While wearing the Myo armband on the upper side of the forearm, participants performed ten ordered activities: (1) reading aloud on a desk, (2) treadmill-walking at the self-selected slow speed, (3) treadmill-walking at the self-selected fast speed, (4) resting on a chair, (5) smoking while sitting, (6) talking over the phone, (7) eating while sitting, (8) smoking while walking and talking, (9) smoking while talking and sitting, and (10) smoking while walking silently. Except for eating and smoking, all activities lasted for the maximum of 5 min. Participants also had an unconstrained break of 10–15 min duration between cigarettes. A freely available mobile application (aTimeLogger-Time Tracker) was employed to record the start and end time information of the performed activities. All data were transferred to a laptop computer via Bluetooth during the study. The dataset contained 25 h of daily activity and 64 smoking events. The smoking events had a total duration of 4.75 h. The remaining data contain different hand-related activities such as cell phone usage (39 times), providing signature in the paper (16 times), holding food plate and drinking cup (52 times), eating and dining activities (more than 100 activities), shaking hands (13 times), holding handrails, doorknobs (more than 50 times), etc. All these hand-related activities were included under the broad category “non-smoking events”. Figure 2 shows the characteristic sensor signals during a smoking session. Figure 2c–e indicates that EMG signals have different patterns for puffing duration.

### 2.3. EMG Channel Selection

A channel selection method was performed to determine the most relevant sEMG channels, as using many sEMG channels is computationally expensive for real-time applications. Furthermore, too many EMG recordings could lead the model to overfit the training instance due to irrelevance or redundancy. In the literature, several methods have been used to determine the most informative sEMG channels including Fisher–Markov selector [36], sequential forward selection (SFS) [37,38], and direct channel selection [39]. In this study, a commonly used SFS-based channel selection algorithm was used. The SFS chooses the best single particular channel for classifier input, and then it adds another channel to it at a time to maximize the accuracy of the classifier in combination with chosen channels.

## 3. Data Processing and Classifier

Encouraged by the excellent performance of Convolutional neural networks (CNN) techniques in various areas [40–42], several deep learning techniques have been utilized for gesture recognition via sEMG [43–45]. In this study, we formulated the cigarette smoking recognition as an image classification problem using a CNN and Long Short-Term Memory (LSTM) framework. To construct an image with a size of H × W × D (height × width × depth), several techniques can be used. In [46–48], the authors used the recorded high-density EMG channel signals as an image, with the dimension of the image at the same height and width as the EMG channel array. In [43,44], a sliding time window was used to segment sEMG signals. In that study, the dimension of the image matches the sensor channel number and the length of the window. Another successful approach uses the Short-Time Fourier Transform (STFT) of segmented signals, with the size of the image being equal to frequency × time × channels [49,50].

**Figure 3.**
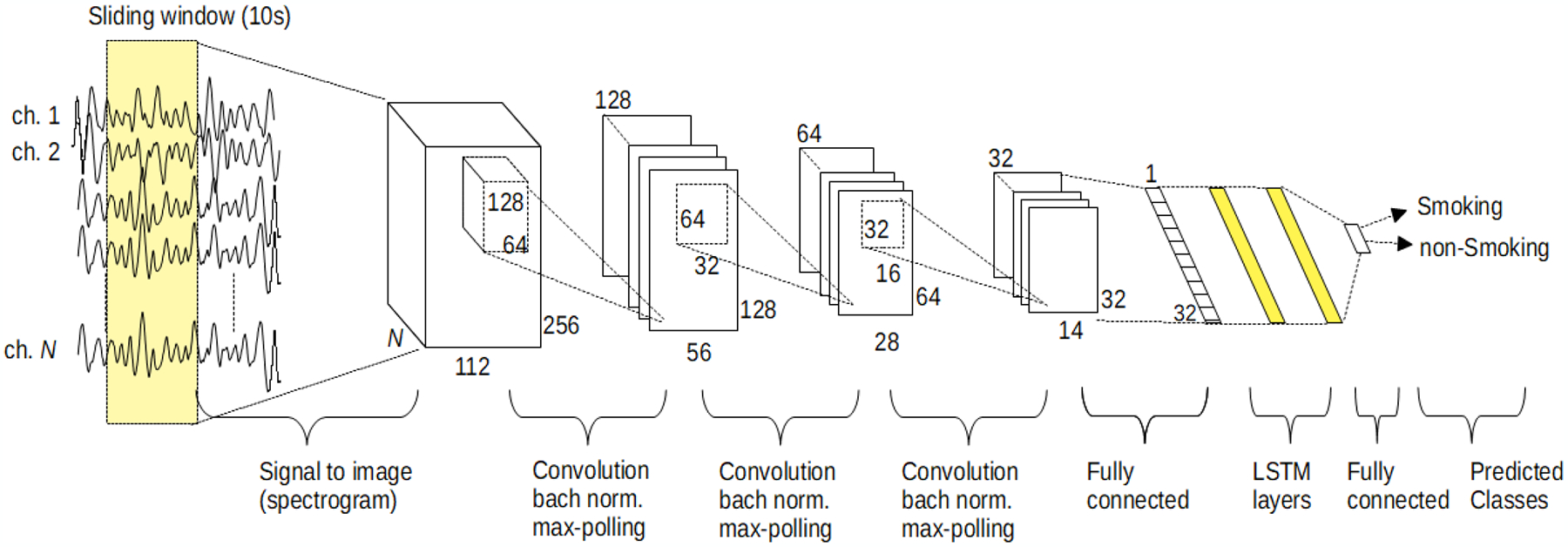
Proposed CNN-LSTM architecture for smoking detection.

In this study, the spectrogram approach was utilized, as informed by [27]. The signal stream was segmented by sliding windows with a length of 10 s and a sliding length of 5 s. we have computed the spectrogram of each segment of the EMG channels by using a 512-point FFT with a Hamming type of window that length of 256 with 95% overlap. Therefore, each segment converted to a matrix (image) of 256 × 112×N (frequency × time bins × channels). By using this approach, the ~25 h time series of EMG signal was converted to more than 18,000 images. The proposed classifier model was composed of two essential neural network groups. The CNN group was responsible for the extraction of features. The duty of the second group was the classify each segmented window as a cigarette smoking activity or not by using an LSTM network. Figure 3 illustrates the simplified structure of the proposed model.

The first stage of the model composed of three two-dimensional convolutional layers, followed by a fully connected layer that has 32 outputs. After each convolutional layer, a batch normalization, a linear rectifier layer, and a max-pooling layer, which find the maximum feature map over two neighborhoods, were used. After the fully connected layer, a dropout layer was utilized with a 0.5 dropout rate for reducing the overfitting risk during the training process. The feature map size of each convolution layer was chosen as 128, 64, and 32 with the motivation of the study [51]. For all convolution layers, the dimension of the filters was assigned as the 0.5 of their input image dimension. To reduce the size of the feature map to 32, we utilized a fully connected layer. In the second group of the proposed model, two sequential LSTM layers (by the motivation of the results in [52]) with the cell size of 64 and one output layer were used to classify the temporal dynamics of the extracted 32 features. The proposed model was trained by using Adam [53] optimizer with a mini-batch size of 64, five iterations, and a learning rate of 10^−2^.

### Performance Measure

The ground truth information obtained from the activity logger application was used to label the class information of each sliding window. If a sliding window corresponded to any cigarette smoking session, it was labeled as the smoking class. Otherwise, it was categorized as a non-smoking class. A leave-one-subject-out (LOSO) cross-validation approach was employed in this study to assure the person-independence of the trained model. In this method, we left out data that belong to one particular subject iteratively from the whole dataset. The data of that left out subject was employed for the evaluation of the model trained in this iteration. The following performance metrics were computed: the false positive (FP), true positive (TP), false negative (FN), recall (Rec), precision (Prec), F1-score (F1), and accuracy (Acc). In the Results section, the accumulated confusion matrices over all subjects are presented.

## 4. Results

**Figure 4.**
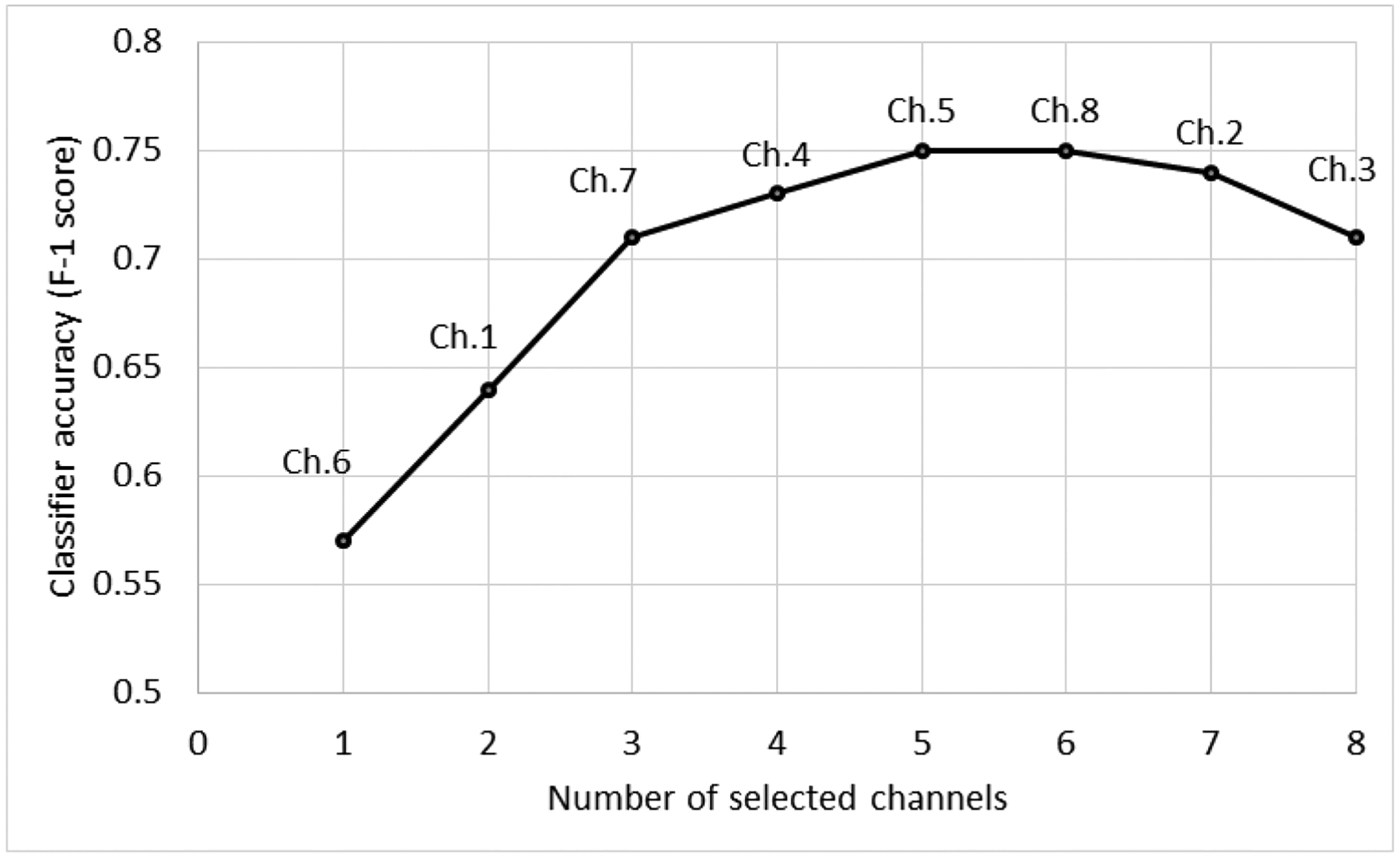
Sequential forward selection results on individual sEMG channels.

The cigarette smoking detection performance of the proposed model for different sensory modalities is presented in Table 3. In the study, we investigated the detection of cigarette smoking hand gestures from sEMG signals. The results show that sEMG signals could be used to track smoking-related hand gestures. The use of sEMG signals provided an F1-score of 0.75, whereas the IMU-based approach reached an F1-score of 0.81 for the detection of cigarette smoking. However, if we use sEMG signals as a secondary sensory modality, the combination of IMU and sEMG approach reached up to an F1-score of 0.84. This result shows that sEMG signals could increase detection performance by about 3%. This increment was statistically significant (McNemar’s test [54]), *p* < 4.9 × 10^−12^. Figure 4 shows the SFS EMG channel selection results for classification performances in each step of SFS. A subset of five channels had a better classification performance than the combination of all eight channels. According to the channel selection, brachioradialis, flexor carpi ulnaris, extensor carpi radialis, and flexor carpi radialis were likely involved in this subset of more effective classifier data. Although the location of sEMG channels was not the same for all subjects, these general groups may provide better detection performance for smoking activity.

## 5. Discussion

This study shows that only using sEMG signals is underperformance compared to a single IMU to recognize cigarette smoking. This performance difference may arise because the collected sEMG signals only monitor finger and hand movement but do not provide information about forearm motions (elbow flexion and elbow extension) as the muscles (biceps brachii, brachialis, and coracobrachialis) that are responsible forearm gestures are located in the upper arm [55]. Using another Myo armband on the upper arm could be a solution. However, this means an extra device which not suitable for long-term studies. The difference may also be due to the fact that the sEMG patterns and sEMG sensor locations vary from person to person. On the other hand, the usage of sEMG signals with an IMU has increased classification performance on cigarette smoking recognition. The previous studies increased this performance by using multiple IMUs on other parts of the arm/body (Table 1). However, multiple device usage is obstructive for smokers and not suitable for free-living studies. In this study, we proposed an approach that allows the motion of the forearm and hand and finger motions with a single device.

This present study has some limits which ought to be considered in the interpretation of its results. The Myo armband has dry electrodes. Compared to gel electrodes, dry electrodes are not accurate enough and less resistant to motion artifacts [56]. Additionally, the Myo armband allows a maximum of 200 Hz sampling rate, which is less than the preferred value of 1 kHz [57]. One of the other limitations of the study is that the Myo armband was not calibrated individually for each participant. At the beginning of the study, the device was calibrated by a researcher, and the same setting was used for all participants. Future work with individual calibration may enhance the detection performance of the armband. One of the other limitations of the study is that the study is performed with limited participants and for a limited duration. As a future study, we are planning to conduct a new study in a free-living condition with higher participants. This study did not investigate optimal hyperparameters for the proposed model. In future research, these parameters could be optimized by using grid search, Gaussian processes, or genetic optimizer.

## 6. Conclusions

In this work, we investigated the use of finger and hand gestures for cigarette smoking detection by measuring sEMG-based muscle activity signals from the forearm. We also evaluated the combination of sEMG-based muscle activity and IMU-based motion for cigarette smoking recognition. The results showed that using only sEMG signals would not provide superior cigarette smoking detection performance relative to IMU signals. However, the measurement of sEMG improved smoking detection results when combined with IMU signals without the necessity of using an additional device. The results are encourage further study on the use of sEMG sensors under free-living conditions. The size of the Myo armband and the placement location on the forearm could be obstructive for some participants. In future research, two or three sEMG electrodes could be located under a wristband for a more compact and comfortable device configuration with the intent to achieve better recognition of daily activities such as drinking, eating, or smoking.

## Figures and Tables

**Table 1.** Summary of the studies employing wearable sensors for smoking monitoring.

Study	Sensor Type	F1-Score	Validation	Dataset	Classifier	Sensor Location (Red) Measurement Area (Blue)
[19]	9D-IMU	0.85	10-fold	15 subjects	RF	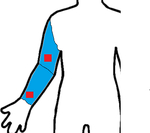
[21]	3D-IMU	0.79	5-fold	6 subjects (11.8 h)	RF	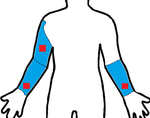
[22]	6D-IMU	0.83–0.94	LOSO	11 subjects (45 h)	Hierarchical	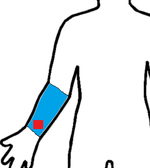
[23]	6D-IMU	0.08–0.86	-	6 subjects (21 h)	SVM, Edge det.	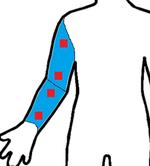
[24]	RIP 6D-IMU	0.91	10-fold	6 subjects (40 h)	SVM	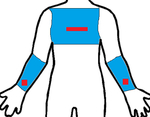
[25]	6D-IMU Smart lighter	0.85	LOSO	35 subjects (816 h)	SVM	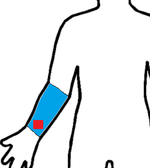
this study	sEMG 6D-IMU	0.84	LOSO	16 subjects (25 h)	CNN-LSTM	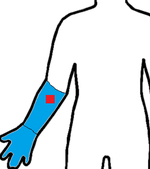

**Table 2.** Possible motions and activated muscles for cigarette smoking.

Motion	Activated Muscles
Elbow flexion (EF)	Biceps brachii, Brachioradialis, Brachialis
Elbow extension (EE)	Anconeus, Triceps brachii
Forearm supination (FS)	Supinator, Biceps brachii (long head)
Forearm pronation (FP)	Pronator quadratus, Pronator teres
Wrist flexion (WF)	Flexor carpi radialis, Flexor carpi ulnaris, Palmaris longus
Wrist extension (WE)	Extensor carpi radialis longus, Extensor carpi radialis brevis, Extensor carpi ulnaris
Tip pinch	Extensor Digitorium, Abductor Pollicis Longus, Flexor Digitorum Profundus

**Table 3.** Confusion matrices and performance metrics of the proposed detection algorithm for different sensor combinations.

	TPs	FPs	FNs	TNs	Rec	Prec	F1	Acc
sEMG (all 8 channels)	4329	1312	1897	10,959	0.69	0.76	0.70	0.82
sEMG (selected channels: 6, 1, 7, 4, 5)	4711	1294	1515	10,977	0.75	0.78	0.75	0.84
IMU	4765	854	1351	11,292	0.79	0.85	0.81	0.87
IMU+sEMG (selected channels)	5091	697	1135	11,574	0.82	0.88	0.84	0.90

## Abbreviations

CNN: Convolutional Neural Network
EMG: Electromyogam
IMU: Inertial Measurement Unit
LOSO: Leave-One-Subject-Out
LSTM: Long Short-Term Memory
SFS: Sequential Feature Selection
STFT: Short-Time Fourier Transform
